# A return to the environment in epidemiology: multilevel analysis of diabetes mellitus in a large urban agglomeration of Argentina

**DOI:** 10.1590/1980-549720260043

**Published:** 2026-08-17

**Authors:** María Jimena Marro, Maria de Jesus Mendes da Fonseca, Iuri da Costa Leite, Christian Ballejo, Marcio Alazraqui

**Affiliations:** I Instituto Nacional de Epidemiología "Dr. Juan H. Jara"-ANLIS Malbrán – Mar del Plata (Buenos Aires), Argentina.; II Fundação Oswaldo Cruz, Escola Nacional de Saúde Pública Sérgio Arouca – Rio de Janeiro (RJ), Brazil.; III Universidad Nacional de Lanús, Institute of Collective Health – Remedios de Escalada (Buenos Aires), Argentina.

**Keywords:** Diabetes mellitus, Green areas, Multilevel analysis, Built environment

## Abstract

**Objective:**

The burden of disease due to diabetes mellitus has shown an increasing trend worldwide. Given the potential of physical structures to create opportunities for the adoption of individual behaviors, this study aimed to analyze the relationship between neighborhood food environments and green spaces and the prevalence of diabetes mellitus in the Mar del Plata-Batán urban agglomeration in Argentina, while also considering individual characteristics, from 2013 to 2018.

**Methods:**

A cross-sectional design with a multilevel approach was used. Individual data from the National Risk Factor Survey were linked to data on food environments and green spaces aggregated at the census tract level. Multilevel logistic regression models were used, with individuals nested within census tracts; odds ratios were estimated for independent variables with 95% confidence intervals.

**Results:**

A total of 946 observations distributed across 81 census tracts were analyzed. After adjustment for individual variables, living in census tracts with a high density of healthy food outlets was associated with a 43% reduction in the odds of having diabetes [OR 0.57 (0.35–0.93)]. Public green space area per inhabitant in the neighborhood was not significantly associated with the odds of diabetes. The proportion of variability attributable to the contextual level ranged from 2.35 to 1.20%.

**Conclusions:**

The neighborhood food environment was associated with diabetes mellitus in the studied agglomeration. These results may encourage the development of longitudinal studies with greater power to demonstrate the causal role of the food environment in the occurrence of diabetes.

## INTRODUCTION

Since the late 20^th^ century, the global burden of disease attributable to diabetes mellitus has increased steadily, underscoring its persistent importance as a major public health concern^1,2^. In Argentina, the prevalence of diabetes increased from 8.4% in 2005 to 12.7% in 2018^3^. This upward trend, together with the increasing prevalence of obesity and other nutrition-related diseases, has occurred in parallel with a global rise in the consumption of ultra-processed foods^4^, further reinforcing the association between dietary patterns and diabetes.

Given the emergence of diabetes as a complex, multidimensional public health challenge^5^, research has historically prioritized individual-level determinants, emphasizing their association with established risk factors such as obesity and physical inactivity^6,7^. However, interest in the role of contextual factors has resurged in recent decades^8-13^. Recognizing that the physical features of the urban environment can shape opportunities for adopting healthy behaviors^14-16^, increasing attention has been directed toward food and recreational environments.

Food production, distribution, and retail systems vary across countries. In the United States, supermarkets have been used as a proxy for healthy food outlets, and greater proximity to these establishments has been inversely associated with the prevalence of obesity and diabetes mellitus^17,18^. In contrast, in countries such as Spain, Brazil, and Mexico, this role has been attributed to fruit and vegetable markets or small local food retailers^19-21^. Furthermore, although exposure to green spaces has been associated with a protective effect against diabetes, the evidence has been less consistent than that for the food environment^22,23^.

Given the limited evidence on this topic in Argentina, this study aimed to examine the association between neighborhood food environments, green spaces, and the prevalence of diabetes mellitus in the Mar del Plata–Batán urban agglomeration during the 2013–2018 period, while accounting for individual-level characteristics.

## METHODS

A cross-sectional study with a multilevel approach was conducted to examine the relationships between contextual and individual characteristics in relation to public health outcomes. This approach enables the simultaneous consideration of factors operating at both the group and individual levels^24^.

The study was conducted in the Mar del Plata-Batán urban agglomeration, one of Argentina's eight largest urban centers and the administrative seat of *Partido de General Pueyrredon* (PGP), in Buenos Aires Province. PGP has a population of 667,082 inhabitants (316,475 men and 350,607 women)^25^. Mar del Plata-Batán has 39.2 km of coastline, a mean annual temperature of 14.1°C, and an average wind speed of 21 km/h^26^.

A two-level multilevel analytical framework was employed, consisting of Level 1 (individual) and Level 2 (census tract). The Mar del Plata-Batán urban agglomeration comprises 879 urban census tracts (geostatistical units with an average of 300 dwellings)^27^, 16 rural census tracts, and three mixed census tracts. For the purposes of this study, census tracts were used as proxies for neighborhoods^28^.

The data source for Level 1 was the National Risk Factor Survey (*Encuesta Nacional de Factores de Riesgo* – ENFR), specifically the 2013 and 2018 editions. Level 2 data were obtained from the following sources: the green space mapping developed by Karis et al., based on data collected in the study area during 2017 and 2018^29^; the database of businesses active as of April 22, 2019, provided by the Municipality of General Pueyrredon (MGP); and the 2010 National Census of Population, Households, and Dwellings^30^, which provided population data at the census tract level.

ENFR was designed to collect information on risk factors and the prevalence of noncommunicable diseases among individuals aged 18 years old and older residing in private households located in Argentine localities with populations of 5,000 or more. The survey employed a probabilistic, multistage sampling design. In the 2018 edition, data collection comprised three components: (1) a self-reported questionnaire, (2) physical and anthropometric measurements, and (3) biochemical assessments. The 2013 edition included only the self-reported questionnaire. The ENFR estimation domains encompassed Argentina's six geographic regions, its 24 jurisdictions, and the eight urban agglomerations with populations of 500,000 or more, including Mar del Plata-Batán^31^. As part of the sampling process conducted by the National Institute of Statistics and Censuses, 83 census tracts were randomly selected within the Mar del Plata-Batán agglomeration.

Consistent with previous studies^32,33^, this study combined data from two editions of the ENFR by merging the 2013 sample and the sample from Step 1 of the 2018 survey, and selecting participants from the Mar del Plata-Batán urban agglomeration. Consequently, all Level 1 variables were based on self-reported information. This approach was adopted to maximize the sample size within each census tract, under the assumption that contextual conditions remained relatively stable over the study period.

The business database is an administrative registry of all businesses registered as active with the MGP (the local government authority for the study area) and includes information on business type and address. The categories "greengrocers/fruit shops" and "health food stores/herbalists" were selected to represent two components of the local food environment: the location of food outlets and the availability of healthy foods^21^. Business addresses were geocoded to obtain their geographic coordinates (latitude and longitude) and were subsequently assigned to census tracts using reverse geocoding based on digital census tract maps.

The green space mapping was developed by the authors^29^ using primary data derived from digital imagery. The indicator selected was "public green space per inhabitant." Public green spaces were defined as publicly accessible, state-owned open areas characterized by a predominance of vegetation and other natural elements, serving functions related to recreation, contact with nature, and social interaction.

The outcome of interest was diabetes mellitus, defined as self-reported elevated blood glucose or a diagnosis of diabetes made by a healthcare professional (yes/no). Level 1 variables included sex (male, female); age (18–24, 25–34, 35–49, 50–64, and ≥65 years); educational attainment (up to incomplete primary education, complete primary and incomplete secondary education, and complete secondary education or higher); physical activity level (high, moderate, low); and average daily fruit and/or vegetable consumption (calculated by multiplying the number of servings consumed by the number of days of consumption during a typical week and dividing the result by seven). The physical activity variable was derived in the ENFR based on the number of days participants engaged in physical activity during the week preceding the interview, as well as the duration and intensity of those activities. Physical activity levels were classified as high, moderate, or low according to the recommendations of the international physical activity questionnaire^31^. Level 2 variables included:

- Food environment: Defined as the density of healthy food outlets, calculated as the ratio of the number of greengrocers/fruit and vegetable markets or health food/herbal stores to the number of inhabitants within the census tract, using a scaling factor of 10,000.
- Green spaces: Defined using the public green space per capita indicator^29^, which measures the total area of public green spaces (neighborhood squares, public squares and urban parks, large parks, and nature reserve areas) in m^2^ per inhabitant. Because this indicator is defined for the nineteen PGP zones (according to the zoning established by the authors), the value for each zone was assigned to the census tracts comprising that zone. This variable was used as a proxy for the recreational environment of the census tract.

The contextual variables were categorized into three-level ordinal variables based on tertiles of their observed distributions, using the following cutoffs: low [0–4.43], medium [4.43–42.50], and high [42.50–851.29].

**Figure 1:**
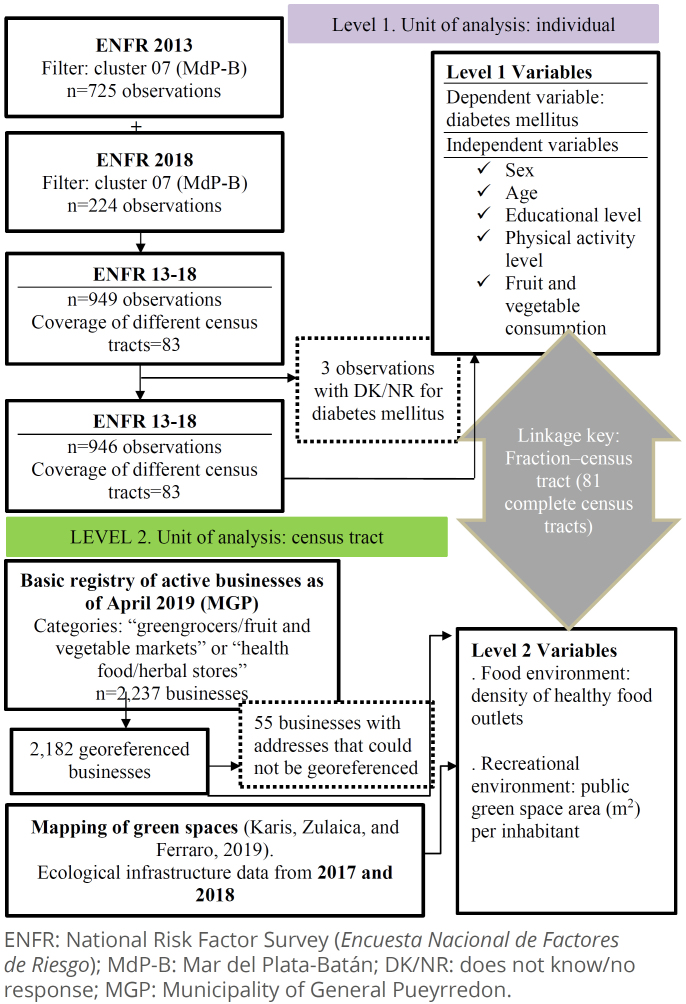
Flow diagram of the data source selection process used in the study according to levels.

**Source:** Prepared by the authors.

The analytical dataset was created by merging the table of individual-level variables from the 2013–2018 ENFR (Urban Agglomeration 7: Mar del Plata-Batán) with a table containing contextual variables, using the census tract identifier as the linking variable (Figure 1).

Categorical variables were summarized using percentage distributions, whereas continuous variables were described using measures of central tendency and dispersion. The complex sampling design of the ENFR was taken into account by applying sampling weights in the estimation of percentages and means. Consistent with the approach adopted in previous studies^20,34,35^, all subsequent analyses examining the associations between participant characteristics and the likelihood of diabetes were performed using the unweighted sample.

Records with "does not know/no response" (DK/NR) values for the dependent variable (n=3) were excluded and were not imputed because they represented a negligible proportion of the observations. Multilevel logistic regression models were fitted with individuals nested within census tracts. Effect estimates were expressed as odds ratios (OR) with corresponding 95% confidence intervals (95%CIs). Initially, a null model consisting of a logistic regression with no explanatory variables and a random intercept at the census tract level was fitted. Univariate models were then constructed for each independent variable to assess its association with the odds of diabetes (unadjusted analysis). Subsequently, two adjusted models were fitted, each including the contextual variable that was significant at the 10 % level (p<0.10). Model 1 included sex and age, whereas Model 2 included all individual-level variables that were statistically significant at the 10% level (p<0.10) in the unadjusted analyses, in addition to sex. In the adjusted models, the category with the largest number of observations was used as the reference category for each variable. Interpretation focused on the contextual variable that remained statistically significant at the 5% level, whereas individual-level variables were included solely as adjustment covariates. Interactions between the individual- and contextual-level variables included in the models were also evaluated.

The general equation of the fitted model was:


$$ln\left(\frac{p_{ij}}{1-p_{ij}}\right)=\overset{`}{\beta }x_{ij}+\overset{´}{\gamma }z_{j}+u_{0j}$$

Where: $ln\left(\frac{p_{ij}}{1-p_{ij}}\right)$ is the natural logarithm of the odds of diabetes mellitus for individual *i* in census tract j; $x_{ij}$ is the vector of individual-level covariates; $Z_{j}$ is the vector of contextual-level covariates; and $u_{0j}$ is the random effect representing the variability in the odds of diabetes across census tracts. The random effect $u_{0j}$ is assumed to follow a normal distribution with a mean of zero and variance $\sigma_{u}^{2}$ .

The variance partition coefficient (VPC) was estimated to quantify the proportion of the total variance attributable to the contextual level (census tracts). Because the individual-level variance cannot be estimated directly in multilevel logistic regression models, a fixed level-1 variance of π^2^/3 was assumed^36,37^. In addition, the pseudo-R^2^ statistic proposed by Snijders and Bosker^37^, which represents the proportion of variance explained by the model, as well as the level-2 pseudo-R^2^, were calculated^38^.

Statistical analyses were performed using R software (version 4.5.0)^39^ and the tidyverse^40^, sf^41^, tmaptools^42^, lme4^43^, and performance^44^ packages. This study is part of the doctoral project in Collective Health at Universidad Nacional de Lanús (UNLa), entitled "Diabetes mellitus in context: Multilevel analysis in an urban agglomeration in Argentina" (*Diabetes mellitus en contexto. Análisis multinivel en un aglomerado urbano de Argentina*), which was approved by the Ethics Committee of UNLa. This article represents a partial output of that research project.

### Data availability statement:

The complete dataset supporting the results of this study is available from the corresponding author upon request. The dataset is not publicly available due to the database integrationperformed by the authors.

## RESULTS

A total of 949 observations were obtained from the 2013 and 2018 editions of the ENFR corresponding to the Mar del Plata-Batán agglomeration (725 in 2013 and 224 in 2018). Three DK/NR records regarding diabetes prevalence were excluded. It was possible to standardize and geolocate 97.5% (n=2,182) of the greengrocers/fruit shops and health food/herbal stores. After merging the Level 1 and Level 2 databases, 946 observations distributed across 81 census tracts remained for analysis (Figure 1).

The highest concentration of diabetes cases was observed among older age groups, with individuals aged 50–64 years representing the most relevant category. The largest proportion of participants had completed at least secondary education. Low levels of physical activity were reported by 59.8% of individuals with diabetes in 2013 and 70.0% in 2018. The median density of healthy food outlets was 17.2 per 10,000 inhabitants, with substantial variation across census tracts: while some tracts had no healthy food outlets, others presented densities of up to 851 outlets. The median area of public green space was 2.8 m^2^ per inhabitant (Table 1).

**Table 1:** Absolute and percentage distribution of individual variables according to the edition of the National Risk Factor Survey, based on the presence of diabetes mellitus, and summary measures of contextual variables, Mar del Plata-Batán, 2013 and 2018.

		2013			2018		
		DM: Yes [weighted count × 1,000 (percentage)]	DM: No [weighted count × 1,000 (percentage)]	p-value	DM: Yes [weighted count × 1,000 (percentage)]	DM: No [weighted count × 1,000 (percentage)]	p-value
Sex							
	Male	30.1 (51.7)	183.6 (45.7)	0.327	43.1 (43.1)	180.7 (47.9)	0.654
	Female	28.1 (48.3)	218.2 (54.3)		56.9 (56.9)	196.2 (52.1)	
Age							
	18–24	0.6 (1.1)*	64.4 (16.0)	<0.001	13.1 (13.1)*	47.9 (12.7)	0.316
	25–34	3.8 (6.5)*	84.3 (21.0)		4.7 (4.7)*	82.3 (21.8)	
	35–49	14.9 (25.6)	102.0 (25.4)		27.2 (27.2)*	97.9 (26.0)	
	50–64	20.9 (35.9)	78.9 (19.6)		32.2 (32.2)	71.5 (19.0)	
	64+	17.9 (30.8)	72.2 (18.0)		22.9 (22.9)	77.0 (20.4)	
Educational level							
	Incomplete primary education or less	7.5 (12.9)	22.4 (5.6)	0.026	7.3 (7.3)*	7.6 (2.0)*	0.269
	Completed primary education and incomplete secondary education	23.4 (40.2)	153.8 (38.3)		39.5(39.5)	123.9 (32.9)	
	Completed secondary education or higher	27.3 (46.9)	225.6 (56.1)		53.2 (53.2)	245.4 (65.1)	
Physical activity level†							
	High	4.7 (8.1)*	77.9 (19.4)	0.034	6.6 (6.6)*	85.5 (22.7)	<0.001
	Moderate	18.6 (32.0)*	132.8 (33.0)		19.5 (19.5)	132.3 (35.1)	
	Low	34.8 (59.8)	188.7 (47.0)		70.0 (70.0)	159.0 (42.2)	
Mean daily fruit and vegetable consumption (number of servings)							
	Mean (SE)	2.3 (0.2)	2.3 (0.1)	0.714	1.9 (0.3)	2.5 (0.2)	0.119
**Contextual variables**		**Median (IQR)**					
	Healthy food outlet density‡ (number of healthy food outlets/number of inhabitantsx10,000)	17.2 (0:5.9)					
	Green spaces§ (m^2^/number of inhabitants in the area)	2.8 (1.1:4.8)					

SE: standard error, IQR: interquartile range; p-value: Pearson's χ² test with Rao & Scott correction or t-test

* estimates are unreliable because they have a coefficient of variation >33.3%

† missing data: 2,400 observations in 2013 and 3,800 observations in 2018

‡ data corresponding to 2019

§ data corresponding to 2017 and 2018.

**Source:** Prepared by the authors.

The odds of having diabetes increased with age: individuals aged 50–64 years had approximately seven times the odds of having diabetes compared with those aged 18–24 years. Among individuals aged 65 years old or older, the odds remained elevated, although it was slightly lower than that observed in the 50–64-year age group. No significant differences were identified according to sex. Individuals with low physical activity levels had 150% higher odds of having diabetes compared with those in the high physical activity group, whereas an educational level above incomplete primary education was associated with a 70% reduction in these odds. Regarding contextual variables, residence in census tracts with a high density of healthy food outlets was inversely associated with the odds of having diabetes (p<0.10). Additionally, the proportion of individuals with diabetes was higher in areas with low or medium densities of healthy food outlets. The density of green spaces at the census tract level was not significantly associated with the odds of having diabetes (Table 2).

**Table 2:** Crude analysis of factors associated with the odds of having diabetes mellitus, considering the random effect, Mar del Plata-Batán, 2013 and 2018.

Characteristics		Crude OR	90%CI	p-value	Diabetes (%)
Individual variables					
Sex					
	Male	1.00			16.2
	Female	0.88	0.65–1.20	0.502	14.4
Age					
	18-24	1.00			4.1
	25-34	1.76	0.66-4.72	0.346	6.9
	35-49	3.53	1.44–8.67	0.021	12.8
	50-64	7.10	2.46–20.45	<0.001	22.7
	64+	6.42	2.24–18.40	<0.001	20.9
Educational level					
	Incomplete primary education or less	1.00			31.9
	Completed primary education and incomplete secondary education	0.36	0.22–0.59	<0.001	14.6
	Completed secondary education or higher	0.32	0.20–0.52	<0.001	13.1
Physical activity level					
	High	1.00			8.3
	Moderate	1.84	1.06–3.20	0.069	14.0
	Low	2.48	1.47–4.17	0.004	18.1
	Fruit and vegetable consumption	0.99	0.90–1.08	0.827	
**Contextual variables**					
Food environment (healthy food outlet density)					
	Low	1.00			16.5
	Moderate	1.09	0.74–1.59	0.718	17.8
	High	0.66	0.43–0.99	0.095	11.5
Green spaces					
	Low	1.00			16.0
	Moderate	0.87	0.59–1.26	0.531	14.3
	High	1.01	0.64–1.60	0.958	16.1

OR: odds ratio; 90%: 90% confidence interval.

**Source:** Prepared by the authors.

After adjustment for individual-level variables, residence in census tracts with a high density of healthy food outlets was associated with a 43% reduction in the odds of having diabetes (Table 3, Model 2). Although the confidence intervals of the estimates were wide, the associations remained statistically significant (p<0.05) (Table 3). Neither the random effects nor the tested interactions reached statistical significance. The proportion of total variability attributable to the contextual level was 2.35% in the model including only the food environment variable (unadjusted model) and 1.20% in the full model (Model 2). Conversely, the proportion of variance explained by the model increased progressively from 1.41% in the unadjusted model to 16.79% in the full model. The proportional reduction in Level 2 variance between the null model and the adjusted models was greater in Model 2 (Table 4).

**Table 3:** Multilevel logistic regression models for the odds of having diabetes mellitus, Mar del Plata-Batán, 2013 and 2018.

		Unadjusted model		Model 1		Model 2	
		OR (95%CI)	p-value	OR (95%CI)	p-value	OR (95%CI)	p-value
Individual variables							
Sex							
	Female			1	-	1	-
	Male			1.18 (0.81–1.73)	0.389	1.19 (0.81–1.76)	0.372
Age							
	64+			1	-	1	-
	50–64			1.09 (0.68–1.74)	0.777	1.16 (0.72–1.88)	0.539
	35–49			0.54 (0.33–0.89)	0.015	0.61 (0.36–1.03)	0.064
	25–34			0.27 (0.13–0.53)	<0.001	0.33 (0.16–0.67)	0.002
	18–24			0.15 (0.05–0.42)	<0.001	0.13 (0.04–0.43)	0.001
Educational level							
	Completed secondary education or higher					1	-
	Completed primary education and incomplete secondary education					0.90 (0.59–1.35)	0.601
	Incomplete primary education or less					1.60 (0.85–3.01)	0.144
Physical activity level							
	Low					1	-
	Moderate					0.79 (0.52–1.20)	0.268
	High					0.50 (0.26–0.95)	0.036
**Contextual variables**							
Food environment (healthy food outlet density)							
	Low	1	-	1	-	1	-
	Moderate	1.09 (0.69–1.72)	0.718	1.04 (0.66–1.64)	0.874	0.94 (0.60–1.49)	0.799
	High	0.66 (0.40–1.08)	0.095	0.58 (0.35–0.95)	0.029	0.57 (0.35–0.93)	0.026
Random effects							
	σ^2^	3.29		3.29		3.29	
	τ_00_	0.11		0.05		0.04	

σ^2^: individual-level variance; τ_00_: contextual-level variance; OR: odds ratio; 95%CI: 95% confidence interval. Unadjusted model: includes only the contextual variable food environment; Model 1: adjusted for sex and age; Model 2: adjusted for sex, age, educational level, and physical activity level.

**Source:** Prepared by the authors.

**Table 4:** Proportion of variability explained by multilevel logistic regression models for the odds of having diabetes mellitus and variance partition coefficients, Mar del Plata-Batán, 2013 and 2018.

Model	Snijders and Bosker pseudo-R^2^ (%)	Level 2 pseudo-R^2^ (%)	VPC (%)
Null model	0.00	0.00	3.80
Unadjusted model	1.41	39.03	2.35
Model 1	13.12	58.44	1.61
Model 2	16.79	69.10	1.20

VPC: variance partition coefficient; Unadjusted model: includes only the contextual variable food environment; Model 1: adjusted for sex and age; Model 2: adjusted for sex, age, educational level, and physical activity level.

**Source:** Prepared by the authors.

## DISCUSION

This study examined the association between selected built environment characteristics and diabetes mellitus in one of Argentina's eight major urban agglomerations. Individuals living in urban census tracts with a higher density of healthy food outlets had lower odds of having diabetes after adjustment for individual-level factors, including age, sex, physical activity, and education.

The findings regarding the association between a healthy food environment and diabetes are consistent with those reported in previous studies^8,11,45^. At the regional level, a study conducted in Minas Gerais, Brazil, found that residence in areas with higher household incomes and a greater density of private facilities for physical activity was associated with lower odds of having diabetes^46^. A study from Mexico demonstrated that adults living in neighborhoods where the density of fruit and vegetable outlets decreased, while the density of chain convenience stores increased, had higher odds of diabetes^20^.

Although this study employed a cross-sectional design, which limits the ability to establish causal associations, the findings are consistent with those from longitudinal studies. A systematic review and meta-analysis including longitudinal designs that investigated the association between built environment factors and the incidence of type 2 diabetes mellitus identified a protective effect of healthy food environments against the development of the disease^47^. The studies cited above did not include built environment data from Argentina. Within the national context, research investigating the relationship between socioeconomic status and noncommunicable diseases has shown that higher neighborhood-level educational attainment was associated with a lower odds of diabetes among women; however, this association was reversed when socioeconomic status was assessed at the city level, while no differences were observed among men^48^. To date, no studies have been identified that analyze the relationship between food environments or green spaces and diabetes mellitus in Argentina.

The substantial transformations in the "macro" dimensions of the food system that have occurred in Latin America in recent decades have altered food environments, with subsequent changes in consumer behaviors that contribute to obesity^49,50^. The mechanisms through which the food environment may influence diabetes are multiple, as it can act as a mediator between the "macro" dimensions of the food system and individual behaviors. In this regard, evidence indicates consistent associations between the availability of fruit and vegetable outlets and higher consumption of these foods^51-53^, which represents a protective factor against the disease.

No association was observed between the extent of green space and diabetes in this study, unlike findings from studies conducted in other countries that reported an inverse association^54,55^, as well as those from a systematic review of longitudinal studies^23^. A study conducted across cities in eleven Latin American countries reported a weak inverse association between green space availability and the likelihood of diabetes^56^. Conversely, differences in the methodologies used to measure green space across studies may influence the observed associations.

Interest in studying neighborhood physical characteristics and their association with health outcomes is based on the premise that these characteristics may influence health-related behaviors by either constraining or facilitating their adoption^15^. Furthermore, this perspective aims to shift the focus from individuals and their "individual choices" toward the contextual conditions that enable specific behaviors. This study also considered Patricia Aguirre's observations^57^ regarding the substantial influence of income on "what can be purchased to eat"; statistically, this aspect was addressed by adjusting for educational attainment as a proxy for income level.

Regarding the limitations of this study, the database selection process was complex and involved the use of data collected in different years. Because two editions of the survey were combined, a certain degree of stability in the outcome variable was assumed, considering the chronic nature of diabetes mellitus. Regarding the data sources for the Level 2 variables, efforts were made to minimize the time interval between the collection periods of these sources and 2018 (the most recent ENFR edition available at the time of the analysis).

Although no specific power calculation was performed for the associations investigated, this study analyzed data representative of the urban population of Mar del Plata-Batán. Given the sample size and the number of higher-level units (946 individuals distributed across 81 census tracts), the study was considered adequately powered to estimate the associations of interest.

On the other hand, self-reported diabetes mellitus has shown a sensitivity of approximately 60%^58,59^. Therefore, it is reasonable to assume that a proportion of individuals with diabetes was not identified by the ENFR. Under the assumption of non-differential misclassification, this limitation may have resulted in an underestimation of the observed association. Furthermore, although the selection of adjustment variables was based on the theoretical model, not all potential confounding factors may have been considered, such as ethnicity, which was not available in the ENFR dataset.

Regarding the use of the multilevel model, although the random effects were not statistically significant, this approach allowed the incorporation of contextual variables into the analysis while accounting for the nested structure of the data. Furthermore, some healthy food outlets operating within the informal economy may not have been captured because they were not included in the business registry. Consequently, the observed measure of association may have been underestimated, and the effect of the food environment may be greater.

Regarding the strengths of this study, the identification of an inverse association between a healthy food environment and diabetes mellitus represents a relevant contribution to the epidemiology of the disease in Latin America, considering that research has focused predominantly on individual-level factors. Furthermore, the importance of investigating local environments, given the variability observed across countries, is emphasized. These findings may encourage the development of longitudinal studies, which provide greater potential to demonstrate the causal role of the food environment in the occurrence of diabetes.

Finally, the need to advocate for improvements in data sources related to contextual factors and their availability for health research in Latin America is recognized as imperative.
