# Supplementary material for: A return to the environment in epidemiology: multilevel analysis of diabetes mellitus in a large urban agglomeration of Argentina
Source: Rev Bras Epidemiol. 2026 Aug 17;29:e260043. doi: 10.1590/1980-549720260043 (PMC13480625; doi:10.1590/1980-549720260043)
Supplement: Translation

# Translation: Un retorno al ambiente en epidemiología: análisis multinivel de la diabetes mellitus en un gran aglomerado urbano de Argentina

**DOI:** 10.1590/1980-549720260043.2

**Authors:** María Jimena Marro, Maria de Jesus Mendes da Fonseca, Iuri da Costa Leite, Christian Ballejo, Marcio Alazraqui

**Affiliations:** I Instituto Nacional de Epidemiología "Dr. Juan H. Jara"-ANLIS Malbrán – Mar del Plata (Buenos Aires), Argentina.; II Fundação Oswaldo Cruz, Escola Nacional de Saúde Pública Sérgio Arouca – Rio de Janeiro (RJ), Brasil.; III Universidad Nacional de Lanús, Instituto de Salud Colectiva – Remedios de Escalada (Buenos Aires), Argentina.

**Keywords:** Diabetes mellitus, Áreas verdes, Análisis multinivel, Entorno construido

## Abstract

**Objetivo:**

La carga de enfermedad por diabetes mellitus muestra una tendencia ascendente a nivel global. Dado el potencial de las estructuras físicas de creación de oportunidades para la adopción de comportamientos individuales, el objetivo fue analizar la relación entre el entorno alimentario y los espacios verdes de los barrios y la prevalencia de diabetes mellitus en el aglomerado Mar del Plata-Batán de Argentina, considerando además características individuales, en 2013-2018.

**Métodos:**

Se utilizó un diseño transversal con enfoque multinivel. Se vincularon datos individuales de la Encuesta Nacional de Factores de Riesgo con datos sobre entorno alimentario y espacios verdes agregados a nivel de radio censal. Se utilizaron modelos de regresión logística multinivel con individuos anidados en radios censales; se estimaron *odds ratio* para las variables independientes con intervalos de confianza del 95%.

**Resultados:**

Se trabajó con 946 observaciones distribuidas en 81 radios censales. Luego de ajustar por variables individuales, residir en radios con alta densidad de comercios de alimentos saludables se asoció con una disminución de la chance de tener diabetes del 43% [OR 0,57 (0,35–0,93)]. La superficie verde pública por habitante del barrio no mostró asociación significativa con la chance de diabetes. La proporción de variabilidad atribuible al nivel contextual fue entre el 2,35 y el 1,20%.

**Conclusiones:**

el entorno alimentario del barrio se asoció con la diabetes mellitus en el aglomerado estudiado. Estos resultados podrían impulsar el desarrollo de investigaciones con diseños longitudinales, de mayor potencia en la demostración del rol causal del entorno alimentario en la ocurrencia de diabetes.

## INTRODUCCIÓN

Desde finales del siglo XX, la carga de enfermedad por diabetes mellitus viene mostrando una tendencia ascendente a nivel global, lo que señala su actualidad como problema de salud colectiva^1,2^. En Argentina, la prevalencia de diabetes aumentó de 8,4% en 2005 a 12,7% en 2018^3^. Este incremento, sumado al de la obesidad y otras enfermedades relacionadas con la nutrición, se produjo paralelamente a un aumento del consumo de alimentos ultraprocesados en diferentes regiones del mundo^4^, reforzando la relación entre alimentación y diabetes.

Considerando la emergencia de la diabetes como problema complejo en múltiples planos^5^, históricamente, hubo un privilegio del nivel individual, resaltando su asociación con factores de riesgo clásicos como obesidad e inactividad física^6,7^. Sin embargo, en las últimas décadas, ha resurgido el interés por el rol de los factores contextuales^8-13^. En función del potencial de las estructuras físicas del ambiente urbano de creación de oportunidades para la adopción de comportamientos individuales^14-16^, se focalizó en los entornos alimentario y recreativo.

Los circuitos de producción, distribución y comercialización de alimentos varían entre países: en Estados Unidos los supermercados fueron considerados un *proxy* de comercios de alimentos saludables, por lo que su cercanía se asoció inversamente a la ocurrencia de obesidad o diabetes mellitus^17,18^, mientras que, en España, Brasil o México, dicho lugar lo ocuparon los negocios de verduras y frutas o pequeños almacenes^19-21^. Por otro lado, si bien se ha demostrado el efecto protector sobre la diabetes de la exposición a espacios verdes, la evidencia ha sido menos contundente que en el caso del entorno alimentario^22,23^.

Dado el escaso desarrollo de investigaciones en la temática en Argentina, el objetivo de este artículo es analizar la relación entre el entorno alimentario y los espacios verdes de los barrios y la prevalencia de diabetes mellitus en el gran aglomerado urbano Mar del Plata-Batán de Argentina, considerando además características individuales, en 2013–2018.

## MÉTODOS

Se utilizó un diseño transversal con enfoque multinivel, que facilita el estudio de las relaciones entre características del contexto y el individuo para eventos de interés en salud pública, posibilitando la consideración de factores tanto del nivel grupal como del individual^24^.

El área de estudio corresponde a Mar del Plata-Batán, uno de los ochos centros urbanos de Argentina y ciudad cabecera del Partido de General Pueyrredon (PGP), provincia de Buenos Aires. El PGP cuenta con una población de 667.082 habitantes (316.475 varones, 350.607 mujeres)^25^. Mar del Plata-Batán posee 39,2 km de costa marina, con una temperatura media anual de 14,1 °C y una velocidad promedio de sus vientos de 21 km/hora^26^.

Se trabajó con una estructura multinivel con dos niveles de análisis: nivel 1 (individual) y nivel 2 (radios censales). El aglomerado Mar del Plata-Batán está compuesto por 879 radios censales urbanos (unidades geoestadísticas con un promedio de 300 viviendas)^27^, 16 rurales y 3 mixtos; en este trabajo fueron considerados como *proxy* de vecindario o barrio^28^.

La fuente de datos para el nivel 1 fue la Encuesta Nacional de Factores de Riesgo (ENFR) en sus ediciones 2013 y 2018. Para el nivel 2 se utilizaron: la cartografía de espacios verdes elaborada por Karis et al. con datos del área de estudio correspondientes a los años 2017 y 2018^29^, la base de datos de comercios activos al 22 de abril de 2019 otorgada por la Municipalidad de General Pueyrredon (MGP) y el Censo Nacional de Población, Hogares y Viviendas 2010^30^ para el dato de la población por radio censal.

Las ENFR buscaron obtener información sobre factores de riesgo y prevalencia de enfermedades no transmisibles en población de 18 años y más, residente en viviendas particulares en localidades con 5.000 o más habitantes de Argentina. El diseño muestral fue probabilístico y multietápico. La edición 2018 incluyó tres pasos: el primero consistió en un relevamiento por autorreporte a partir de un cuestionario, el segundo incorporó mediciones físicas y antropométricas y el tercero, parámetros bioquímicos. La encuesta del año 2013 solo utilizó autorreporte. Los dominios de estimación de la ENFR fueron las seis regiones de Argentina, las 24 jurisdicciones y los ocho aglomerados urbanos de 500.000 habitantes o más, entre los que se encuentra Mar del Plata-Batán^31^. En función del proceso muestral realizado por el Instituto Nacional de Estadística y Censos, en este último aglomerado fueron seleccionados aleatoriamente 83 radios censales.

En esta investigación, al igual que lo hicieron otros estudios^32,33^, se combinaron dos ediciones de la ENFR: la muestra del paso 1 del año 2018 y la del 2013, seleccionando el aglomerado Mar del Plata-Batán en ambas, por lo que todas las variables del nivel 1 fueron por autorreporte. Esta combinación tuvo el objetivo de maximizar el tamaño muestral para cada radio censal, asumiendo cierta estabilidad en las condiciones del contexto.

La base de datos de comercios es un registro administrativo de todos los comercios inscriptos en la MGP (entidad de gobierno del área de estudio) en condición de activos, con datos del rubro y domicilio. Se seleccionaron los rubros "verdulerías/fruterías" o "dietéticas/herboristerías", considerando dos de los componentes del entorno alimentario local: ubicación y disponibilidad de alimentos saludables^21^. Los domicilios fueron georreferenciados obteniendo su latitud y longitud, y luego identificados en cada radio censal mediante geocodificación inversa espacial a través de cartografía digital de radios censales.

La cartografía de espacios verdes fue elaborada por las autoras^29^ a partir de fuentes primarias con imágenes digitales. Se tomó el indicador "superficie verde pública por habitante". Las áreas verdes públicas fueron definidas como espacios abiertos donde predomina la cobertura vegetal y los elementos naturales, de acceso libre, perteneciente al Estado y con funciones de recreación, contacto con la naturaleza e interacción social.

El desenlace de interés fue la diabetes mellitus, definida como el auto reporte de azúcar alta en sangre o de diagnóstico de diabetes por parte de un profesional de la salud (sí, no). Las variables del nivel 1 fueron: sexo (varón, mujer); edad (18–24, 25–34, 35–49, 50–64, 65 y más años); nivel de instrucción (hasta primario incompleto, primario completo y secundario incompleto, secundario completo y más); nivel de actividad física (alto, medio, bajo) y promedio de consumo diario de frutas y/o verduras (obtenido a partir de la multiplicación de la cantidad de porciones por la cantidad de días en que se consumieron en una semana típica, dividido por 7). La variable nivel de actividad física fue construida en la ENFR en función de la cantidad de días en que la persona realizó actividad física en la semana previa a la entrevista, tiempo e intensidad de la actividad, categorizados según recomendaciones del cuestionario internacional de actividad física en alto, medio y bajo^31^. Las variables del nivel 2 fueron:

- Entorno alimentario: definido como la densidad de comercios saludables, calculada como el cociente entre el número de verdulerías/fruterías o dietéticas/herboristerías y el número de habitantes del radio censal^30^, utilizando un factor de ampliación de 10.000.
- Espacios verdes: definida a partir del indicador superficie verde pública por habitante^29^ que mide la extensión del área verde pública total (plazas barriales, plazas y parques urbanos, grandes parques y áreas de reserva natural) en m^2^ sobre el número de habitantes del área. Dado que el dominio del indicador corresponde a las diecinueve zonas del PGP (en función de la zonificación definida por las autoras), el valor de la zona se asignó a los radios constitutivos de cada una de ellas. Esta variable se utilizó como *proxy* del entorno recreativo del radio censal.

Las variables contextuales fueron transformadas en categóricas ordinales de tres niveles: bajo [0–4,43], medio (4,43–42,50] y alto (42,50–851,29], utilizando como puntos de corte los terciles de la distribución observada.

La tabla de datos se construyó uniendo la tabla de variables individuales de la ENFR 2013–2018 (aglomerado 7: Mar del Plata-Batán) y una tabla con las variables contextuales, mediante el identificador fracción-radio censal (Figura 1).

**Figura 1:** Diagrama de flujo del proceso de selección de las fuentes de datos utilizadas en la investigación según niveles.

Fuente: Elaboración propia.

Las variables categóricas fueron descriptas a partir de su distribución porcentual y las continuas por medio de medidas de tendencia central y dispersión. Se tuvo en cuenta el diseño muestral complejo de la ENFR, por lo que se incluyeron ponderaciones en el cálculo de porcentajes y medias. Todos los análisis posteriores, que buscaron encontrar asociaciones entre diversas características y la probabilidad de tener diabetes, se llevaron a cabo con la muestra no ponderada, al igual que lo hicieron otros trabajos^20,34,35^.

Los registros con el valor "no sabe/no contesta" (Ns/Nc) en la variable dependiente (n=3) fueron excluidos y no se imputaron, dado que representaron una proporción pequeña de las observaciones. Se utilizaron modelos de regresión logística multinivel con individuos anidados en radios censales. Los coeficientes estimados se expresaron en términos de razón de momios (*odds ratio*), con sus intervalos de confianza del 95%. En primer lugar, se ajustó un modelo de regresión logística sin variables explicativas y con intercepto aleatorio a nivel de radio censal (modelo nulo). Seguidamente, se construyeron modelos univariados para cada una de las variables independientes con el objetivo de analizar su influencia sobre la chance (odds) de tener diabetes (análisis no ajustado). Luego, se ajustaron dos modelos con la variable contextual que resultó significativa al nivel del 10 % (p<0,10), uno junto con sexo y edad (Modelo 1) y otro con todas las variables individuales estadísticamente significativas al nivel del 10% (p<0,10) en los modelos no ajustados, incluyendo además la variable sexo (Modelo 2). En los modelos ajustados, para cada variable, se tomó como categoría de referencia aquella con el mayor número de observaciones. Se interpretó la variable contextual que resultó estadísticamente significativa al nivel del 5%; las variables individuales se consideran sólo para el ajuste. Se pusieron a prueba las interacciones entre las variables individuales y contextuales del modelo.

La ecuación general del modelo ajustado fue:


$$ln\left(\frac{p_{ij}}{1-p_{ij}}\right)=\overset{`}{\beta }x_{ij}+\overset{´}{\gamma }z_{j}+u_{0j}$$

Donde: $ln\left(\frac{p_{ij}}{1-p_{ij}}\right)$ $ es el logaritmo neperiano de la chance de tener diabetes mellitus para el individuo *i* en el radio j; $x_{ij}$ es el vector de covariables a nivel individual; $Z_{j}$ es el vector de covariables a nivel contextual; y $u_{0j}$ es el parámetro aleatorio que mide la variabilidad entre los radios censales en la chance de tener diabetes. Se asume que $u_{0j}$ sigue una distribución normal con media cero y varianza $\sigma_{u}^{2}$ .

Se estimó el coeficiente de partición de la varianza (VPC, por sus siglas en inglés) para cuantificar la proporción de la variabilidad total atribuible al nivel contextual (radios censales). Dado que en modelos logísticos la varianza del nivel individual no es directamente estimable, se asumió una varianza fija de π^2^/3 para el nivel 1^36,37^. Además, se calculó el pseudo-R^2^ propuesto por Snijders y Bosker^37^, que corresponde a la proporción de la varianza explicada por el modelo, y el pseudo-R^2^ del nivel 2^38^.

Para el análisis estadístico se utilizó el software R (versión 4.5.0)^39^, paquetes tidyverse^40^, sf^41^, tmaptools^42^, lme4^43^ y performance^44^. Esta investigación integra el proyecto del doctorado en Salud Colectiva (UNLa) titulado "Diabetes mellitus en contexto. Análisis multinivel en un aglomerado urbano de Argentina", que fue aprobado por el Comité de Ética de la Universidad Nacional de Lanús, siendo este artículo un producto parcial.

### Declaración sobre la disponibilidad de datos:

El conjunto completo de datos que respalda los resultados de este estudio está disponible previa solicitud al autor correspondiente. El conjunto de datos no está disponible públicamente debido a la integración de la base de datos realizada por los autores.

## RESULTADOS

Se obtuvieron 949 observaciones de las ediciones 2013 y 2018 de la ENFR correspondientes al aglomerado Mar del Plata-Batán (725 en 2013, 224 en 2018); fueron eliminados tres registros Ns/Nc en la prevalencia de diabetes. Fue posible normalizar y geolocalizar el 97,5% (n=2.182) de las verdulerías/fruterías y dietéticas/herboristerías. Luego de la unión de las bases de datos de los niveles 1 y 2, quedaron para el análisis 946 observaciones distribuidas en 81 radios censales (Figura 1).

La mayor concentración de casos de diabetes se observó en los grupos etarios avanzados, siendo el intervalo de 50 a 64 años el más relevante. El grupo con nivel de instrucción de secundario completo y más fue el mayoritario. El 59,8% de las personas con diabetes en 2013 y el 70,0% en 2018 realizaba bajo nivel de actividad física. La mediana de densidad de comercios de alimentos saludables fue 17,2 cada 10.000 habitantes con amplia dispersión: mientras algunos radios no registraron comercios saludables, otros tuvieron una densidad de 851. La mediana de superficie verde pública fue de 2,8 m^2^ por habitante (Tabla 1).

**Tabla 1:** Distribución absoluta y porcentual de las variables individuales según edición de la Encuesta Nacional de Factores de Riesgo en función de la presencia de diabetes mellitus y medidas resumen de las variables contextuales, Mar del Plata-Batán, 2013 y 2018.

		2013			2018		
		DM: Si [conteo ponderado x 1.000 (porcentaje)]	DM: No [conteo ponderado x 1.000 (porcentaje)]	p-valor	DM: Si [conteo ponderado x 1.000 (porcentaje)]	DM: No [conteo ponderado x 1.000 (porcentaje)]	p-valor
Sexo							
	Varón	30,1 (51,7)	183,6 (45,7)	0,327	43,1 (43,1)	180,7 (47,9)	0,654
	Mujer	28,1 (48,3)	218,2 (54,3)		56,9 (56,9)	196,2 (52,1)	
Edad							
	18–24	0,6 (1,1)*	64,4 (16,0)	<0,001	13,1 (13,1)*	47,9 (12,7)	0,316
	25–34	3,8 (6,5)*	84,3 (21,0)		4,7 (4,7)*	82,3 (21,8)	
	35–49	14,9 (25,6)	102,0 (25,4)		27,2 (27,2)*	97,9 (26,0)	
	50–64	20,9 (35,9)	78,9 (19,6)		32,2 (32,2)	71,5 (19,0)	
	64+	17,9 (30,8)	72,2 (18,0)		22,9 (22,9)	77,0 (20,4)	
Nivel de instrucción							
	Hasta primario incompleto	7,5 (12,9)	22,4 (5,6)	0,026	7,3 (7,3)*	7,6 (2,0)*	0,269
	Primario completo y secundario incompleto	23,4 (40,2)	153,8 (38,3)		39,5(39,5)	123,9 (32,9)	
	Secundario completo y más	27,3 (46,9)	225,6 (56,1)		53,2 (53,2)	245,4 (65,1)	
Nivel de actividad física†							
	Alto	4,7 (8,1)*	77,9 (19,4)	0,034	6,6 (6,6)*	85,5 (22,7)	<0,001
	Medio	18,6 (32,0)*	132,8 (33,0)		19,5 (19,5)	132,3 (35,1)	
	Bajo	34,8 (59,8)	188,7 (47,0)		70,0 (70,0)	159,0 (42,2)	
Promedio de consumo diario de frutas y verduras (N° porciones)							
	Media (EE)	2,3 (0,2)	2,3 (0,1)	0,714	1,9 (0,3)	2,5 (0,2)	0,119
**Variables contextuales**		**Mediana (RIQ)**					
	Densidad de comercios saludables‡ (N° comercios saludables/N° habitantesx10.000)	17,2 (0:5,9)					
	Espacios verdes§ (m^2^/N° habitantes del área)	2,8 (1,1:4,8)					

EE: error estándar, RIQ: rango intercuartílico; p-valor: χ^2^ Pearson con corrección Rao & Scott o t test

* estimaciones no confiables por presentar coeficiente de variación >33,3%

† sin dato: 2.400 observaciones en 2013 y 3.800 observaciones en 2018

‡ datos correspondientes al año 2019

§ datos correspondientes a los años 2017 y 2018.

Fuente: Elaboración propia.

La chance de tener diabetes aumentó con la edad: la chance de las personas de 50 a 64 años fue aproximadamente siete veces la de aquellas con edad entre 18 y 24 años; en el grupo de 65 años o más, la chance se mantuvo elevada, aunque ligeramente inferior a la observada en 50 a 64 años. No se hallaron diferencias significativas por sexo. El grupo con bajo nivel de actividad física tuvo una chance de tener diabetes 150% mayor al de nivel alto, mientras que el tener un nivel de instrucción superior al de primario incompleto disminuyó dicha chance en un 70%. Con relación a las variables contextuales, el vivir en radios censales con alta densidad de comercios de alimentos saludables se asoció de manera inversa con la chance de tener diabetes (p<0,10); además, la proporción de personas con diabetes fue más alta en entornos con densidad de comercios saludables baja o media. La densidad de espacios verdes de los radios censales no mostró asociación significativa con la chance de tener diabetes (Tabla 2).

**Tabla 2:** Análisis bruto de los factores asociados a la chance de tener diabetes mellitus considerando el efecto aleatorio, Mar del Plata-Batán, 2013 y 2018.

Variables		OR bruto	IC90%	p-valor	Diabetes (%)
Individuales					
Sexo					
	Varón	1,00			16,2
	Mujer	0,88	0,65–1,20	0,502	14,4
Edad					
	18-24	1,00			4,1
	25-34	1,76	0,66-4,72	0,346	6,9
	35-49	3,53	1,44–8,67	0,021	12,8
	50-64	7,10	2,46–20,45	<0,001	22,7
	64+	6,42	2,24–18,40	<0,001	20,9
Nivel de instrucción					
	Hasta primario incompleto	1,00			31,9
	Primario completo y secundario incompleto	0,36	0,22–0,59	<0,001	14,6
	Secundario completo y más	0,32	0,20–0,52	<0,001	13,1
Nivel de actividad física					
	Alto	1,00			8,3
	Medio	1,84	1,06–3,20	0,069	14,0
	Bajo	2,48	1,47–4,17	0,004	18,1
	Consumo de frutas y verduras	0,99	0,90–1,08	0,827	
**Contextuales**					
Entorno alimentario (densidad de comercios saludables)					
	Baja	1,00			16,5
	Media	1,09	0,74–1,59	0,718	17,8
	Alta	0,66	0,43–0,99	0,095	11,5
Espacios verdes					
	Bajo	1,00			16,0
	Medio	0,87	0,59–1,26	0,531	14,3
	Alto	1,01	0,64–1,60	0,958	16,1

OR: odds ratio; IC90%: intervalo de confianza del 90%.

Fuente: Elaboración propia.

Luego de ajustar por variables individuales, residir en radios censales con alta densidad de comercios de alimentos saludables se asoció con una disminución de la chance de tener diabetes del 43% (Tabla 3, Modelo 2). Si bien los intervalos de confianza de las estimaciones fueron amplios, las asociaciones fueron estadísticamente significativas (p<0,05) (Tabla 3). Los efectos aleatorios no fueron estadísticamente significativos, al igual que las interacciones testeadas. La proporción de la variabilidad total atribuible al nivel contextual fue del 2,35% en el modelo que solo incluyó la variable entorno alimentario (modelo no ajustado) y del 1,20% en el completo (Modelo 2). Por otro lado, la proporción de la varianza explicada por el modelo aumentó progresivamente de 1,41% en el modelo no ajustado al 16,79% en el modelo completo. La reducción proporcional de la varianza del nivel 2 entre el modelo nulo y los ajustados fue mayor con el modelo 2 (Tabla 4).

**Tabla 3:** Modelos de regresión logística multinivel para la chance de tener diabetes mellitus, Mar del Plata-Batán, 2013 y 2018.

		Modelo no ajustado		Modelo 1		Modelo 2	
		OR (IC95%)	p-valor	OR (IC95%)	p-valor	OR (IC95%)	p-valor
Individuales							
Sexo							
	Mujer			1	-	1	-
	Varón			1,18 (0,81–1,73)	0,389	1,19 (0,81–1,76)	0,372
Edad							
	64+			1	-	1	-
	50–64			1,09 (0,68–1,74)	0,777	1,16 (0,72–1,88)	0,539
	35–49			0,54 (0,33–0,89)	0,015	0,61 (0,36–1,03)	0,064
	25–34			0,27 (0,13–0,53)	<0,001	0,33 (0,16–0,67)	0,002
	18–24			0,15 (0,05–0,42)	<0,001	0,13 (0,04–0,43)	0,001
Nivel de instrucción							
	Secundario completo y más					1	-
	Primario completo y secundario incompleto					0,90 (0,59–1,35)	0,601
	Hasta primario incompleto					1,60 (0,85–3,01)	0,144
Nivel de actividad física							
	Bajo					1	-
	Medio					0,79 (0,52–1,20)	0,268
	Alto					0,50 (0,26–0,95)	0,036
**Contextual**							
Entorno alimentario (densidad de comercios saludables)							
	Baja	1	-	1	-	1	-
	Media	1,09 (0,69–1,72)	0,718	1,04 (0,66–1,64)	0,874	0,94 (0,60–1,49)	0,799
	Alta	0,66 (0,40–1,08)	0,095	0,58 (0,35–0,95)	0,029	0,57 (0,35–0,93)	0,026
Efectos aleatorios							
	σ^2^	3,29		3,29		3,29	
	τ*00*	0,11		0,05		0,04	

σ^2^: varianza del nivel individual; τ*00*: varianza del nivel contextual; OR: odds ratio; IC95%: intervalo de confianza del 95%. Modelo no ajustado: incluye solo la variable contextual entorno alimentario; Modelo 1: ajustado por sexo y edad; Modelo 2: ajustado por sexo, edad, nivel de instrucción y nivel de actividad física.

Fuente: Elaboración propia.

**Tabla 4:** Proporción de la variabilidad explicada por los modelos de regresión logística multinivel para la chance de tener diabetes mellitus y coeficientes de partición de la varianza, Mar del Plata-Batán, 2013 y 2018.

Modelo	Pseudo-R^2^ Snijders y Bosker (%)	Pseudo-R^2^ nivel 2 (%)	VPC (%)
Modelo nulo	0,00	0,00	3,80
Modelo no ajustado	1,41	39,03	2,35
Modelo 1	13,12	58,44	1,61
Modelo 2	16,79	69,10	1,20

VPC: coeficiente de partición de la varianza; Modelo no ajustado: incluye solo la variable contextual entorno alimentario; Modelo 1: ajustado por sexo y edad; Modelo 2: ajustado por sexo, edad, nivel de instrucción y nivel de actividad física.

Fuente: Elaboración propia.

## DISCUSIÓN

Esta investigación se focalizó en la asociación entre ciertas características del entorno construido y la diabetes mellitus en uno de los ocho grandes aglomerados urbanos de Argentina. Encontramos que las personas que viven en radios urbanos con mayor densidad de comercios de alimentos saludables mostraron una menor chance de tener diabetes, controlando por la influencia de factores individuales como edad, sexo, actividad física y escolaridad.

Los hallazgos sobre la relación entre entorno alimentario saludable y diabetes son consistentes con trabajos previos^8,11,45^. A nivel regional, un estudio en Minas Gerais, Brasil, encontró que vivir en áreas con altos ingresos familiares y alta densidad de lugares para la práctica de actividad física del ámbito privado, se asociaron a una menor chance de tener diabetes^46^. Un trabajo de México demostró que los adultos de vecindarios que experimentaron una disminución en la densidad de negocios de frutas y verduras y un aumento simultáneo en la de negocios de conveniencia de cadena, tuvieron mayores probabilidades de diabetes^20^.

Si bien esta investigación utilizó un diseño transversal, lo que limita la posibilidad de concluir sobre asociaciones causales, nuestros resultados son consistentes con lo demostrado en estudios longitudinales: una revisión sistemática y metaanálisis que incluyó este tipo de diseños para analizar asociaciones entre factores del ambiente construido y la incidencia de diabetes mellitus tipo 2 encontró un efecto protector de los entornos alimentarios saludables sobre la ocurrencia de la enfermedad^47^. Los trabajos citados no incluyeron datos del entorno construido de Argentina; en el ámbito local, encontramos una investigación que indagó la relación entre el nivel socioeconómico y enfermedades no transmisibles en el país, observando que grados altos de educación a nivel de barrios se asociaron a una menor chance de diabetes en mujeres, mientras que esta relación fue inversa cuando se midió a nivel de ciudades; por otro lado, no hallaron diferencias en varones^48^. A la fecha, no hemos encontrado investigaciones que analicen la relación entre entorno alimentario o espacios verdes y diabetes mellitus en Argentina.

Las importantes transformaciones en las dimensiones "macro" del sistema alimentario suscitadas en América Latina en las últimas décadas generaron una alteración de los entornos alimentarios con la consiguiente modificación de los comportamientos de los consumidores, conducentes a la obesidad^49,50^. Las razones por las cuales el entorno alimentario influenciaría sobre la diabetes son varias, pudiendo actuar como mediador entre las dimensiones "macro" del sistema y los comportamientos de las personas. En este sentido, existe evidencia sobre asociaciones consistentes entre la disponibilidad de negocios de frutas y verduras y un mayor consumo de esos alimentos^51-53^, lo que constituye un factor protector de la enfermedad.

Nuestros resultados no mostraron relación entre la magnitud de los espacios verdes y la diabetes, a diferencia de estudios de otros países que hallaron una asociación inversa^54,55^ al igual que una revisión sistemática de estudios longitudinales^23^. Un trabajo que incluyó ciudades de once países de América Latina reportó una asociación inversa, de baja magnitud, entre los niveles de espacios verdes y la chance de diabetes^56^. Por otro lado, la variación en la metodología de medición de los espacios verdes entre los trabajos podría influir en el hallazgo de asociaciones.

El interés por el estudio de las características físicas de los barrios y su asociación con resultados de salud se fundamenta en que estas podrían actuar por medio de restricciones o mejoras en los comportamientos relacionados con la salud^15^. Por otro lado, este conocimiento intenta descentrar el foco del individuo y sus "elecciones individuales" hacia las condiciones del contexto que habilitan la adopción de ciertos comportamientos. En este trabajo se tuvo en cuenta además lo planteado por Patricia Aguirre^57^ respecto del fuerte condicionamiento de los ingresos sobre "lo que se puede comprar para comer"; esto fue considerado, en términos estadísticos, al ajustar por nivel de instrucción como *proxy* de nivel de ingresos.

Como limitaciones de esta investigación, el proceso de selección de bases de datos fue complejo y utilizó datos provenientes de diferentes años. Al haberse combinado dos ediciones de encuestas, se asumió cierta estabilidad en la variable resultado dada la condición crónica de la diabetes mellitus. En cuanto a las fuentes de datos para las variables del nivel 2, se procuró que la distancia temporal con el año 2018 (última edición de la ENFR) al momento del análisis fuera la menor posible.

Si bien no se realizó un cálculo de poder específico para las asociaciones investigadas, este estudio analizó datos con representatividad para la población urbana de Mar del Plata-Batán. Considerando el tamaño de la muestra y el número de unidades de nivel superior (946 individuos distribuidos en 81 radios censales), entendemos que presenta condiciones adecuadas para la estimación de las asociaciones de interés.

Por otro lado, el autorreporte de la diabetes mellitus mostró una sensibilidad cercana al 60%^58,59^, por lo que cabe considerar que existiría una proporción de la población con diabetes que no ha sido captada por la ENFR. Asumiendo un error de clasificación no diferencial, este efecto podría haber generado una subestimación de la asociación encontrada. Asimismo, si bien la inclusión de las variables de ajuste se basó en el modelo teórico, es factible que no hayan sido considerados todos los posibles factores de confusión, por ejemplo, la variable étnica, que no estaba disponible en la base de datos de la ENFR.

Respecto de la utilización del modelo multinivel, si bien los efectos aleatorios no resultaron estadísticamente significativos, esta elección permitió incorporar al análisis el efecto de variables contextuales respetando la estructura anidada de los datos. Además, algunos comercios de alimentos saludables del circuito de la economía informal pueden no haber sido captados al no estar registradas en el básico de comercios, por lo que la medida de asociación encontrada podría estar subestimada y el efecto del entorno alimentario ser mayor.

Entre las fortalezas, pensamos que el hallazgo de la relación inversa entre el entorno alimentario saludable y la diabetes mellitus representa una contribución relevante a la epidemiología de la enfermedad en América Latina, dado la casi exclusividad de investigaciones del nivel individual. Además, se destaca la importancia de estudiar los entornos locales en función de las variaciones entre países. Estos resultados podrían impulsar el desarrollo de investigaciones con diseños longitudinales, de mayor potencia en la demostración del rol causal del entorno alimentario en la ocurrencia de diabetes.

Finalmente, reconocemos que resulta imperante abogar por una mejoría de las fuentes de datos sobre factores contextuales y su disponibilidad para la investigación en salud en América Latina.
